# Enhancing Dental Pulp Stem Cell Proliferation and Odontogenic Differentiation with Protein Phosphatase 1-Disrupting Peptide: An In Vitro Study

**DOI:** 10.3390/cells13131143

**Published:** 2024-07-03

**Authors:** Anna Kobrock, Bárbara Matos, Daniela Patrício, Liliana Grenho, John Howl, Margarida Fardilha, Pedro S. Gomes

**Affiliations:** 1 Signal Transduction Laboratory, Institute of Biomedicine–iBiMED, University of Aveiro, 3810-193 Aveiro, Portugal; annakobrock@ua.pt (A.K.); barbaracostamatos@ua.pt (B.M.); dmpatricio@ua.pt (D.P.); mfardilha@ua.pt (M.F.); 2 BoneLab-Laboratory for Bone Metabolism and Regeneration, Faculty of Dental Medicine, University of Porto, 4200-393 Porto, Portugal; lgrenho@fmd.up.pt; 3 LAQV/REQUIMTE, Faculty of Dental Medicine, University of Porto, 4200-393 Porto, Portugal; 4 Research Institute in Healthcare Science, University of Wolverhampton, Wolverhampton WV1 1LY, UK; j.howl@wlv.ac.uk

**Keywords:** dental pulp stromal cells (DPSCs), odontogenic differentiation, regenerative endodontics, protein phosphatase 1 (PP1), bioportide

## Abstract

The reparative and regenerative capabilities of dental pulp stem cells (DPSCs) are crucial for responding to pulp injuries, with protein phosphatase 1 (PP1) playing a significant role in regulating cellular functions pertinent to tissue healing. Accordingly, this study aimed to explore the effects of a novel cell-penetrating peptide Modified Sperm Stop 1-MSS1, that disrupts PP1, on the proliferation and odontogenic differentiation of DPSCs. Employing MSS1 as a bioportide, DPSCs were cultured and characterized for metabolic activity, cell proliferation, and cell morphology alongside the odontogenic differentiation through gene expression and alkaline phosphatase (ALP) activity analysis. MSS1 exposure induced early DPSC proliferation, upregulated genes related to odontogenic differentiation, and increased ALP activity. Markers associated with early differentiation events were induced at early culture time points and those associated with matrix mineralization were upregulated at mid-culture stages. This investigation is the first to document the potential of a PP1-disrupting bioportide in modulating DPSC functionality, suggesting a promising avenue for enhancing dental tissue regeneration and repair.

## 1. Introduction

Dental pulp plays a fundamental role in the maintenance of the tooth’s physiological equilibrium. Despite its protection by surrounding mineralized tissues, it is vulnerable to trauma or infection, which may lead to irreversible pulpitis or necrosis [1]. Current therapeutic strategies for these pulp conditions broadly rely on root canal therapy (RCT), which encompasses the removal of the injured pulp, canal disinfection, and the subsequent filling with a biocompatible material. Despite its satisfactory functionality, RCT aims for restorative outcomes, averting the regeneration of the loss/damaged functionality of the pulp [2]. Accordingly, regenerative endodontics has emerged as an innovative therapy, grounded in tissue-engineering principles, to regain functionality—as healing capability, immune-responsiveness, and nociception—of the damaged pulp structures [3].

Dental pulp stem cells (DPSCs) were the first dental-related stem cell population to be isolated and characterized, evidencing the typical characteristics of mesenchymal stem cells [4,5]. They are highly proliferative and have self-renewal capabilities and multilineage differentiation potential. DPSCs may also differentiate into odontogenic and neurovascular lineages, giving rise to dentin-pulp complex-like structures upon in vivo implantation [4,5]. Physiologically, DPSCs play a key role in the homeostatic modulation of the pulp, including the response to tissue injury, which has fostered the exploration of their therapeutic potential in regenerative approaches [6]. The regenerative potential of DPSCs seems to elapse from signal modulation via distinct mechanisms, including the delivery of growth factors, genetic modification, and physical stimulation, processes that can activate distinct signaling pathways [7].

Recent advances have highlighted the critical role of protein phosphatase 1 (PP1) in dentinogenesis [8]. PP1 expression, verified in odontoblasts and precursor populations throughout tooth development, is increased during in vitro odontoblastic differentiation, substantiating the deterministic importance of PP1 in the frame of dental pulp regenerative strategies [9]. In this context, PP1 modulation has been recognized to play a determinant role in distinct cellular functions, including those related to regenerative outcomes [10]. PP1 is an extensively expressed phosphatase, active in serine/threonine residues and part of the phosphoprotein phosphatase (PPP) family of the eukaryotic protein phosphatome [11]. PP1 presents a narrow and strongly regulated substrate selectivity, playing a crucial regulatory role in processes such as cell cycle progression, pre-mRNA splicing and transcription, and protein synthesis [12], being further associated with a modulatory activity within tissue/organ development [13]. Its association with the cell differentiation process, particularly within mesenchymal lineages, indicates a significant modulatory activity in the regenerative framework [14,15,16].

The strategic modulation of PP1 activity presents a novel therapeutic avenue, notably through the application of disrupting peptides [17,18]. Recently, MSS1, a bioactive cell-penetrating peptide also known as a bioportide, has shown promise in disrupting PP1 interactions, thereby modulating enzyme activity. This approach has been demonstrated in spermatozoa, where MSS1 application led to increased PP1 activity and consequent inhibition of sperm motility [19]. Despite its established effects in other cells/tissues, the application of MSS1 in dental pulp stromal cells represents an unexplored approach. Given the peptide’s expected potential to modulate PP1 activity across various tissues, we propose investigating its effects on dental pulp stromal cells’ functionality, hypothesizing that MSS1′s regulatory capabilities on PP1 might offer new insights into dental tissue regeneration strategies.

Accordingly, this work aims to characterize the potential modulatory activity of the MSS1 bioportide in DPSCs functionality, detailing the metabolic activity, proliferation, morphological arrangement, and markers of odontogenic differentiation through gene expression analysis and alkaline phosphatase activity.

## 2. Materials and Methods

### 2.1. Cell Culture

Third-passage DPSCs (Human Dental Pulp Stem Cells, Catalog#:PT-5025, Lonza, Basel, Switzerland) were cultured (10^4^ cells/cm^2^) in alpha-modified Eagle’s medium (α-MEM) supplemented with 10% fetal bovine serum (FBS), 100 IU/mL penicillin, 100 µg/mL streptomycin, and 2.5 µg/mL amphotericin B (basal medium; all reagents from Gibco, Waltham, MA, USA). The cultures were grown in 24-well plates (Falcon, New York, NY, USA) at 37 °C and in a 5% CO_2_ atmosphere. After three days, the culture medium was switched to an odontogenic medium composed of the basal medium supplemented with 10 nM dexamethasone, 50 µg/mL ascorbic acid, and 10 mM β-glycerophosphate (all from Sigma-Aldrich, St. Louis, MO, USA) to induce the odontogenic phenotype [20].

For PP1 activation, at days 7, 14, and 21, cells were exposed to 1 µM of MSS1 in an odontogenic medium without FBS. Prior to MSS1 exposure, cells underwent a 24-h period of FBS deprivation to minimize potential interactions between the bioportide and FBS proteins. A control setup was also maintained where cells were subjected to serum deprivation without MSS1 exposure (FBS -/- condition). In parallel, a positive control was continued where DPSCs were kept in a fully FBS-supplemented odontogenic medium. Twenty-four hours after MSS1 exposure, the cultures were assessed for metabolic activity, cell proliferation, morphological changes, gene expression, and alkaline phosphatase activity. To evaluate gene expression, DPSCs were cultured at a density of 5 × 10^3^ cells/cm^2^ in 6-well plates under the conditions described above.

### 2.2. Metabolic Activity and Cell Proliferation

The metabolic activity of the cultured cells was determined with the alamarBlue^®^ (resazurin) assay (Invitrogen, Carlsbad, CA, USA). Resazurin is a blue dye that is reduced by metabolically active and viable cells into resorufin, a pink/red fluorescent byproduct. After 7, 14, and 21 days of culture, the culture medium was removed, and the cell layer was rinsed with PBS and incubated with the 1× alamarBlue^®^ solution at 37 °C for 3 h. The fluorescence of resorufin was then determined (excitation: 540 nm, emission: 590 nm) in a microplate reader (Synergy HT, Biotek, Winooski, VT, USA) with Gen5 1.09 Data Analysis Software.

Cell proliferation was estimated by measuring the total DNA content using the Quant-iT PicoGreen DNA assay (Invitrogen), according to the manufacturer’s instructions. Cultures were washed with PBS and solubilized with 0.1% (*v*/*v*) Triton X-1000 solution. Cell lysates were mixed with the PicoGreen solution and incubated in the dark at room temperature for 5 min. The fluorescence intensity (excitation: 485 nm, emission: 528 nm) was measured with a microplate reader (Synergy HT, Biotek, Winooski, VT, USA).

### 2.3. Immunostaining of F-Actin Cytoskeleton and Nucleus

After 7, 14, and 21 days, the cell cultures were rinsed with PBS and fixed with a 3.7% formaldehyde solution for 10 min at room temperature. Subsequently, cells were permeabilized with a 0.1% (*v*/*v*) Triton X-100 solution for 15 min. This was followed by incubation with 1% bovine serum albumin (BSA) for 30 min (Sigma-Aldrich, St. Louis, MO, USA) to block non-specific binding sites. The cytoskeletal F-actin was stained with Alexa Fluor^®^ 488 phalloidin (diluted 1:100, incubated 30 min, Molecular Probes, Eugene, OR, USA), and the nuclei were stained with Hoechst dye (8 µg/mL, incubated for 10 min, Enzo, New York, NY, USA). Images of the stained cells were acquired using the Celena S digital imaging system (Logos Biosystems, Anyang, Republic of Korea).

### 2.4. Real-Time Quantitative Polymerase Chain Reaction

Cultures were characterized by a real-time quantitative polymerase chain reaction (RT-qPCR) to assess the expression of relevant odontogenic genes at 7, 14, and 21 days of culture. Total RNA was extracted using the TRIzol™ reagent (Invitrogen, Waltham, MA, USA) and reverse-transcribed into complementary DNA (cDNA) with the NZY First-Strand cDNA Synthesis Kit (Nzytech, Lisbon, Portugal). The expression of the target genes was quantitatively determined on RTPCR equipment (CFX96, BioRad) using iQTM SYBR^®^ Green Supermix (BioRad, Hercules, CA, USA). All genes were normalized to the reference gene (*GADPH*), and relative quantification of gene expression was computed using the 2^−ΔΔCt^ method. All genes are described in Table 1.

### 2.5. Alkaline Phosphatase (ALP) Activity and Cytochemical Staining

ALP activity was evaluated in cell lysates (Triton X-100 0.1%, 30 min) after 7, 14, and 21 days of culture by the hydrolysis of p-nitrophenyl phosphate (p-NPP 25 mM, Sigma-Aldrich, St. Louis, MO, USA) in an alkaline buffer (pH 10.3, 37 °C, 1 h). The reaction was stopped with NaOH 5 M, and the product (p-nitrophenol) was measured at λ = 400 nm in a microplate reader (Synergy HT, Biotek, Winooski, VT, USA). Results were normalized to DNA content and expressed as nano-moles of p-nitrophenol per microgram of protein (nmol/µg protein). Cytochemical staining was conducted in fixed cultures (glutaraldehyde 1.5%, 15 min) upon the incubation of sodium naphthyl phosphate (2 mg/mL, Sigma-Aldrich) and Fast Blue RR (2 mg/mL, Sigma-Aldrich) in Tris buffer (0.1 M, pH 10, 1 h). Stained cultures were observed by light microscopy (Primo Vert™ Inverted Microscope, Carl Zeiss, Jena, Germany).

### 2.6. Statistical Analysis

Experiments were performed in triplicate as independent experiments; all quantitative data were expressed as mean values ± standard deviation. Statistical analysis was performed using the IBM^®^ SPSS^®^ Statistics 25. Data normality was assessed by the Shapiro–Wilk test. Regarding normal datasets, a one-way analysis of variance (ANOVA) was performed, followed by the post hoc Tukey test. For non-parametric datasets, the Kruskal-Wallis test was performed, followed by multiple comparisons using Dunn’s tests. For both, *p*-values ≤ 0.05 were considered significant.

## 3. Results

### 3.1. Metabolic Activity and Cell Proliferation of DPSCs Exposed to MSS1

**Figure 1:**
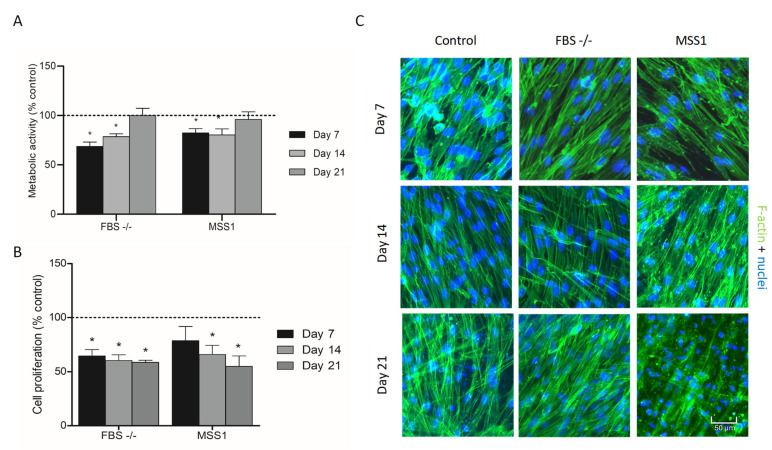
(**A**) Metabolic activity, (**B**) cell proliferation, and (**C**) immunostaining of F-actin cytoskeleton (green) and nuclei (blue) in DPSC cultures subjected to different treatment conditions: full FBS supplementation (Control), FBS deprivation (FBS -/-), and FBS deprivation coupled with MSS1 peptide exposure at 1 µM (MSS1), for 7, 14, and 21 days. * Significantly different from the positive control group, *p* < 0.05. For panels A and B, control values are normalized to 100%; FBS -/- and MSS1 conditions are evaluated relative to this benchmark.

Metabolic activity assays revealed that serum-deprived DPSC cultures exhibited significantly reduced values on days 7 and 14, as compared to the positive control, recovering to levels akin to the positive control by day 21. Similarly, cultures exposed to MSS1 mirrored the trend observed in FBS-deprived cultures (Figure 1A). Regarding proliferation, FBS deprivation notably diminished DPSC proliferation throughout the culture period. In contrast, exposure to MSS1-induced cell proliferation, particularly at early culture stages, achieved proliferation levels on par with the positive control at day 7 (Figure 1B). In addition, cell morphology and general culture organization were evaluated through fluorescence microscopy following F-actin staining and nuclear counterstaining. Within the positive control, cells exhibited an elongated, spindle-shaped, fibroblastic-like morphology, with an increase in cell density and a gradual, directional organization along the cellular long axis over time (Figure 1C)**.** Cultures subjected to FBS deprivation or MSS1 treatment did not display significant differences in morphology or organizational patterns compared to the positive control.

### 3.2. Gene Expression and ALP Activity of DPSCs Exposed to MSS1

**Figure 2:**
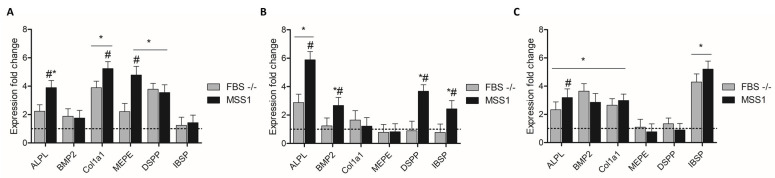
Odontogenic gene expression analysis of DPSCs upon (**A**) 7, (**B**) 14, and (**C**) 21 days of culture, subjected to different treatment conditions: full FBS supplementation (Control), FBS deprivation (FBS -/-), and FBS deprivation coupled with MSS1 peptide exposure at 1 µM (MSS1). * Significantly different from the positive control group, # Significantly different from the FBS -/- group, *p* < 0.05. Control values are normalized to 1; FBS -/- and MSS1 conditions are evaluated relative to this benchmark.

Gene expression analysis of early-stage cultures (day 7) (Figure 2A) revealed a general trend for increased expression of all genes under FBS-deprived conditions, with COL1A1 and DSPP showing significant upregulation. The exposure to MSS1 further induced the expression of ALPL, COL1A1, and MEPE, while the expression profiles of other genes remained comparable to those observed in FBS-deprived conditions. At day 14 (Figure 2B), FBS deprivation significantly upregulated ALPL expression. MSS1 exposure not only significantly increased the expression of ALPL, but also of BMP2, DSPP, and IBSP, as compared to the control and FBS-deprived conditions. Lastly, at day 21, in FBS-deprived conditions, the expression of ALPL, BMP2, COL1A1, and IBSP was increased. Notably, MSS1 treatment led to a significant upsurge in ALPL expression, surpassing levels observed under FBS deprivation (Figure 2C).

**Figure 3:**
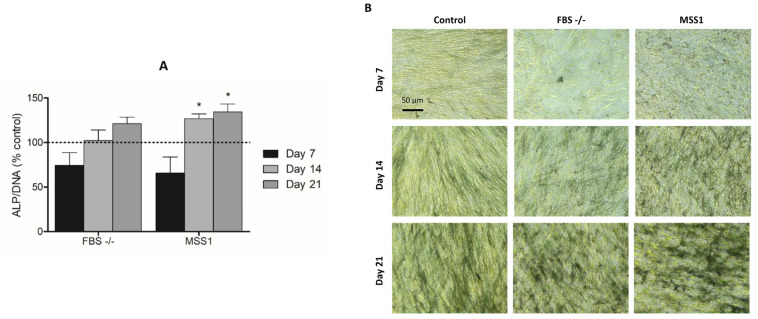
(**A**) Evaluation of ALP activity and (**B**) cytochemical staining of DPSCs subjected to different treatment conditions: full FBS supplementation (Control), FBS deprivation (FBS -/-), and FBS deprivation coupled with MSS1 peptide exposure at 1 µM (MSS1), for 7, 14, and 21 days. * Significantly different from the positive control group, *p* < 0.05. For panel A, control values are normalized to 100%; FBS -/- and MSS1 conditions are evaluated relative to this benchmark.

In evaluating alkaline phosphatase (ALP) activity, cultures subjected to FBS deprivation displayed enzymatic activity levels comparable to those of the positive control, with a noted tendency for enhanced activity over the culture period. The addition of MSS1 resulted in significantly elevated ALP activity compared to the positive control on both days 14 and 21 (Figure 3A). This increase was further supported by cytochemical staining results, which highlighted MSS1-treated cultures as exhibiting the most pronounced staining patterns on days 14 and 21, corroborating the quantitative findings (Figure 3B).

## 4. Discussion

Within the field of dental healthcare, current therapeutic strategies for dental caries predominantly target a restorative outcome and symptomatic relief, often leading to subsequent complications, such as secondary infections, tooth fragility, and increased fracture risk [21]. This situation highlights a pressing need for novel therapeutic strategies that go beyond reparative interventions to enable the regeneration of dental tissues. Against this backdrop, our study introduces a pioneering approach centered on signal modulation through the application of a PP1-disrupting peptide (MSS1). By leveraging the potential of MSS1 to enhance PP1-mediated signaling pathways, we aim to augment cellular functionality within dental pulp stromal cells (DPSCs), offering a promising avenue for advancing regenerative endodontic therapies. This exploration of MSS1′s utility in dental tissue regeneration is particularly timely, as it aligns with the broader goal of shifting from traditional symptomatic treatments to innovative, biology-based solutions that address the underlying causes of dental diseases and facilitate tissue repair and regeneration.

MSS1, designed to mimic the PP1-binding motif RVxF found in approximately 90% of PP1 interactors, including AKAP4—a sperm-specific protein—demonstrates a remarkable capacity to translocate across cell membranes, targeting intracellular domains [22]. This bioportide has been previously demonstrated to selectively disrupt PP1 complexes and enhance PP1 signaling pathways, a functionality initially confirmed in sperm-cell studies [19], marking its first exploration in dental pulp stem cells (DPSCs) within this study. DPSCs were selected for their relevance in prospective regenerative endodontic applications. In order to prime the odontogenic phenotype, cells were grown in the presence of ascorbic acid, dexamethasone, and β-glycerophosphate, substances collectively acknowledged to support this process [20]. Furthermore, to mitigate unforeseen interactions between MSS1 and fetal bovine serum (FBS)-derived proteins, a 24-h serum starvation protocol was implemented before introducing MSS1, with a parallel assessment of serum-deprived controls to discern the specific effects attributable to MSS1 exposure.

In this study, serum deprivation led to a decrease in both the metabolic activity and proliferation of DPSCs without significantly altering cell morphology or the overall organization of the culture. This phenomenon reflects the intricate cell- and time-specific responses elicited by serum starvation [23], a condition that introduces cellular stress due to a nutritionally deficient microenvironment, with effects further varying according to the specific experimental starvation/deprivation settings [24]. Previous research in pulp-derived cells has shown that long-term serum deprivation triggers cell cycle arrest in the S-phase, associated with low cyclin levels and high expression of cyclin inhibitors [25], a pattern consistent with our observations. The exposure to MSS1 was found to induce cell proliferation at early culture stages. Given PP1′s role in modulating various phases of the cell cycle through its specific substrates, its selective inhibition typically results in cell cycle arrest, which can be reversed through PP1 overexpression or inhibition of an antagonistic kinase [26]. Therefore, it is conceivable that the enhancement of PP1 activity by MSS1 alleviates these checkpoint arrests, thereby fostering cell cycle progression and, ultimately, stimulating cell proliferation [27].

MSS1 exposure augmented both odontogenic gene expression and ALP activity. Notably, early culture periods showed a marked increase in COL1A1 and MEPE gene expression. COL1A1 codes for one of the type I collagen subunits, the main structural and most abundant protein in teeth structure [28], establishing a template for the sustenance of cellular proliferation, differentiation, and extracellular matrix mineralization [29]. MEPE codes for matrix extracellular phosphoglycoprotein, an early marker of odontogenic differentiation that is associated with both proliferation and early phase differentiation events [30], despite its potential role in late-stage odontogenesis [31]. At mid-term stages of the DPSC cultures (14 days), MSS1 exposure was found to have induced the expression of DSPP and IBSP. DSPP codes for dentin sialophosphoprotein, a dentin matrix interactive noncollagenous protein with calcium-binding ability, crucial for mineral placement determination and the promotion of odontogenic differentiation [32]. IBSP codes for the integrin-binding sialoprotein, a member of the phosphorylated dentin matrix proteins and a regulator of the extracellular mineral concentrations and matrix mineralization [33,34]. At the late-differentiation stage of the cultures (day 21), MSS1 exposure significantly induced the expression of ALPL, coding for ALP, an induction verified from day 7 onwards. Further assessment through ALP activity and cytochemical staining corroborated this trend, with increased levels being attained with MSS1 exposure. Whether ALP is regarded as an early marker of odontoblastic differentiation, its activity stimulates matrix mineralization by providing phosphates for the dentin biomineralization process, thus being essential at late-differentiation stages [35].

This orchestration of gene expression and functional activity by MSS1 is indicative of its profound effect on PP1 signaling, enhancing odontogenic differentiation through an expected concerted action on various biological targets. This may stem from MSS1′s influence on PP1 signaling over multiple signaling pathways such as the Bone Morphogenetic Protein (BMP) pathway, Wnt/β-Catenin pathway, MAPK/ERK pathway, and PI3K/Akt pathway, all known to modulate cell growth and differentiation in regenerative contexts [36].

## 5. Conclusions

In conclusion, the exposure of DPSCs to MSS1 initiated a rapid stimulation of cell proliferation and a phased activation of the odontogenic differentiation program. This process was characterized by the selective enhancement of PP1-related signaling pathways, which facilitated a temporal stimulation of odontogenesis-related genes; those associated with early differentiation were upregulated at early culture time points, while those associated with matrix mineralization were induced at later time points. Such findings underscore the adeptness of MSS1-modulated signaling to cope with the physiological odontogenic differentiation framework, further inducing a time-specific upregulation of targeted genes, showcasing its potential therapeutic relevance on PP1 signal modulation in regenerative endodontics. Whilst further studies are envisaged to disclose the mechanistic regulatory framework, this investigation reports the first evidence that a PP1-disrupting bioportide can effectively stimulate both the proliferation and odontogenic differentiation of human DPSCs, offering a glimpse into novel therapeutic strategies for dental tissue regeneration.

## Figures and Tables

**Table 1:** Genes and respective primers assay ID (BioRad) for RT-qPCR.

Gene	Assay ID
Glyceraldehyde-3-phosphate dehydrogenase (*GAPDH*)	qHsaCED0038674
Alkaline phosphatase (*ALPL*)	qHsaCED0045991
Bone morphogenic protein-2 (*BMP-2*)	qHsaCID0015400
Collagen type I alpha I chain (*Col1α1*)	qHsaCED0043248
Dentin sialo phosphoprotein (*DSPP*)	qHsaCED0002962
Integrin binding sialoprotein *(IBSP*)	qHsaCED0002933
Matrix extracellular phosphoglycoprotein (*MEPE*)	qHsaCED0045573

## Data Availability

All data generated or analyzed during this study are included in this published article.
